# An SNR Enhancement Method for Φ-OTDR Vibration Signals Based on the PCA-VSS-NLMS Algorithm

**DOI:** 10.3390/s24134340

**Published:** 2024-07-04

**Authors:** Xiaojuan Chen, Haoyu Yu, Jingyao Xu, Funan Gao

**Affiliations:** 1 School of Electronic Information Engineering, Changchun University of Science and Technology, Changchun 130000, China; 2020200100@mails.cust.edu.cn; 2 Shenyang Institute of Engineering, Shenyang 110136, China; 2021623403@stu.sie.edu.cn; 3 State Grid Changchun Electric Power Surrly Company, Changchun 130000, China; ccgaofn@jl.sgcc.com.cn

**Keywords:** PCA-VSS-NLMS, Φ-OTDR, optical fiber sensing, SNR

## Abstract

To improve the signal-to-noise ratio (SNR) of vibration signals in a phase-sensitive optical time-domain reflectometer (Φ-OTDR) system, a principal component analysis variable step-size normalized least mean square (PCA-VSS-NLMS) denoising method was proposed in this study. First, the mathematical principle of the PCA-VSS-NLMS algorithm was constructed. This algorithm can adjust the input signal to achieve the best filter effect. Second, the effectiveness of the algorithm was verified via simulation, and the simulation results show that compared with the wavelet denoising (WD), Wiener filtering, variational mode decomposition (VMD), and variable step-size normalized least mean square (VSS-NLMS) algorithms, the PCA-VSS-NLMS algorithm can improve the SNR to 30.68 dB when the initial SNR is −1.23 dB. Finally, the PCA-VSS-NLMS algorithm was embedded into the built Φ-OTDR system, an 11.22 km fiber was measured, and PZT was added at 10.19–10.24 km to impose multiple sets of fixed-frequency disturbances. The experimental results show that the SNR of the vibration signal is 8.77 dB at 100 Hz and 0.07 s, and the SNR is improved to 26.17 dB after PCA-VSS-NLMS filtering; thus, the SNR is improved by 17.40 dB. This method can improve the SNR of the system’s position information without the need to change the existing hardware conditions, and it provides a new scheme for the detection and recognition of long-distance vibration signals.

## 1. Introduction

The distributed fiber-optic sensor known as the Φ-OTDR system primarily measures phase changes resulting from fiber vibration or stress [1]. This system boasts a long detection range, exceptional sensitivity, and a rapid response time [2], making it a popular choice for applications such as monitoring oil and gas pipelines [3], assessing the structural health of buildings [4], ensuring perimeter security [5], monitoring power and communication safety [6], sensing rail transit systems [7,8], and monitoring engineering geology [9,10].

Utilizing the Φ-OTDR system involves examining the Rayleigh backscattering (RBS) phase alterations within optical fibers. This RBS, being a feeble signal of minimal intensity [11], becomes susceptible to various environmental disturbances, such as polarization fading from the laser itself, interference fading, and external noise. The resulting accumulation of noise within the phase signal, coupled with a diminished signal-to-noise ratio (SNR), ultimately impedes the precision and range capabilities of the sensing operation [12].

One way to enhance hardware design was suggested by Zhu et al. in 2015. These authors introduced an active compensation approach using laser frequency scanning and cross-correlation calculation to mitigate the impact of light source frequency drifting (LSFD). This method effectively detected disturbances at a frequency of 0.5 Hz up to a distance of 5 km along the sensing fiber [13]. Subsequently, in 2016, Baker C et al., proposed a technique utilizing sinusoidally modulated optical signals (SMOSs) to produce high extinction analog pulses. This innovation minimized noise in the backscattering trajectory of the fiber-under-test (FUT) pulses, thus enhancing the signal-to-noise ratio (SNR) of the Φ-OTDR system [14]. Furthermore, Wang et al., presented a novel approach in 2019 that implemented linearization and Golay pulse coding for heterodyne Φ-OTDR. Through experimental validation, they achieved submeter-level measurement accuracy and nano-strain resolution with a sensing range of 10 km [15]. In 2020, Chen et al., proposed a technique involving forced carrier recombination to enhance the extinction ratio (ER) of the semiconductor optical amplifier (SOA). This resulted in a 5.2 dB increase in the SNR of the Φ-OTDR system [12]. Lastly, Li et al., proposed a strategy in 2023 that integrated multi-mode fiber (MMF) and optimized the “n” elastomer to enhance the Φ-OTDR system. This method led to improvements of 10.51 dB and 13.38 dB in position and frequency SNR enhancement, respectively [16].

One way to improve the Φ-OTDR system is to enhance data processing efficiency. Wavelet denoising methods were initially introduced by Qin et al., in 2012 [17], which was followed by the implementation of continuous wavelet transform denoising techniques in the Φ-OTDR system [18]. Wu et al. introduced a trajectory denoising approach for the Φ-OTDR system in 2015 that leveraged multi-scale wavelet decomposition [19]. Moreover, He et al., made strides in 2017 with an adaptive image restoration algorithm employing two-dimensional bi-lateral filtering to boost the SNR of the Φ-OTDR system. By applying this approach to a 27.6 km sensing fiber, the SNR for position information of a signal with an original SNR of 6.43 dB notably increased by more than 14 dB [20]. Lv et al., made further advancements in 2019 by introducing the empirical mode decomposition (EMD) method to address phase drift issues. Notably, when faced with low-frequency external vibrations at 0.5 Hz or 0.3 Hz, the SNR post-phase signal elimination reached impressive levels of 55.58 dB and 44.44 dB [21]. Building upon these innovations, He et al. presented the complete ensemble empirical mode decomposition with adaptive noise (CEEEMDAN) algorithm in 2020, which achieved a significant 52.11 dB increase in the SNR of disturbed positions [22]. In 2021, Jiang et al., introduced a signal enhancement technique for the Φ-OTDR system utilizing deep learning, resulting in an increase in the mean SNR from 13.4 dB to 42.8 dB [23]. Similarly, in 2021, Ma et al., proposed the VSS-NLMS denoising method which enabled the detection of five sets of vibration events ranging from 100 Hz to 500 Hz at the 10.14 km sensing fiber location. The position information SNR for the VSS-NLMS noise reduction approach was enhanced to 59.31 dB, 46.81 dB, 50.14 dB, 34.00 dB, and 67.09 dB [24]. A method based on kurtosis parameter statistical analysis (k-parameter) was introduced by He et al. in 2022, resulting in an improvement in the SNR at 11.9 km on a 12 km fiber to 5.61 dB [25]. Lastly, Liu et al., proposed a genetic least mean square (GLMS) method in 2023, which demonstrated a notable improvement in the SNR ranging from 14.37 dB to 23.60 dB during monotone-scale audio signal testing at 60~1000 Hz. Nevertheless, this method faces challenges such as a low SNR and limited adaptability, leading to subpar denoising effects [26]. In 2023, Turov et al., proposed to present a nonlinear two-dimensional processing method for distributed acoustic sensor data in both time and frequency domains, with an 8-fold increase in operation speed and an improvement in signal-to-noise ratio of 3.7 dB when used alone, and 10.8 dB when used in combination with moving averaging–moving differential (MA-MD) [27].

In order to further improve the vibration signal SNR of the Φ-OTDR system, we proposed a noise reduction algorithm based on PCA-VSS-NLMS to filter the background noise of the heterodyne coherent Φ-OTDR system. In this study, the mathematical principle of the PCA-VSS-NLMS algorithm was constructed in detail, and simulation experiments were performed to verify the filtering effect of the algorithm. The simulation results show that, compared with the WD, Wiener, VMD, and VSS-NLMS algorithms, the PCA-VSS-NLMS algorithm can increase the SNR to 30.68 dB when the initial SNR is −1.23 dB. The proposed algorithm was embedded into the Φ-OTDR system, an 11.22 km fiber was measured, and multiple sets of fixed-frequency disturbances were imposed by adding PZT at 10.19 to 10.24 km. At 100 Hz and 0.07 s, the SNR of the vibration signal is 8.77 dB. After PCA-VSS-NLMS filtering, the SNR is improved to 26.17 dB; thus, the SNR is improved by 17.40 dB. We verified the feasibility of the proposed method from the perspectives of theoretical research, simulation analysis, and laboratory construction and experimentation. By applying the proposed algorithm, the SNR of vibration signals can be improved without changing the existing hardware conditions, which provides a new idea for long-distance vibration signal recognition.

## 2. Working Principle of PCA-VSS-NLMS

**Figure 1:**
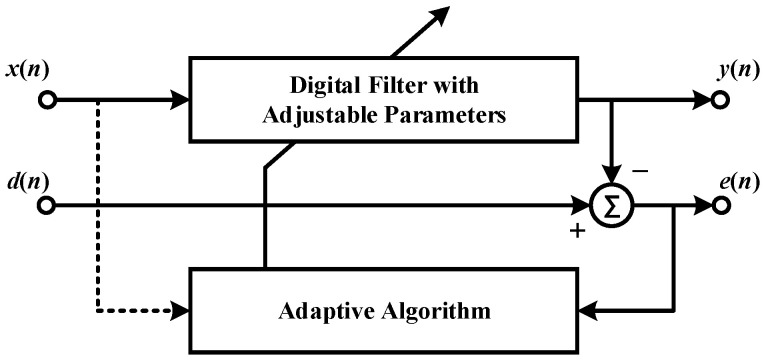
Working principle of adaptive filtering.

An automatically adaptive filter can adjust its parameters based on the input signal’s characteristics to accommodate signal fluctuations. This enhances its ability to process signals that are not consistent over time [28]. When comparing their structures, the normalized LMS (NLMS) algorithm mirrors the standard LMS algorithm [29]. As depicted in Figure 1, both are finite impulse response (FIR) filters, with the weight controller mechanism being the distinguishing factor. The output signal M×1 generated by the input signal x(n) of the y(n) tap will be subtracted from the expected signal d(n) to obtain the error signal e(n). In response to the combined action of the input signal x(n) and error signal e(n), weight adjustment to the weight controller is applied to the FIR filter. In a large number of adaptive loops, the weight vector of the filter is adjusted repeatedly until the filter reaches a steady state.

The basic principle of the NLMS algorithm is that $\hat{w}\left(n\right)$ represents the old weight vector of the filter during the adaptive cycle n and $\hat{w}\left(n+1\right)$ represents the new weight vector of the filter during the adaptive cycle n+1. Then, the design criterion of the NLMS algorithm can be expressed as a constrained optimization problem [30]:

(1) $$\delta \hat{w}\left(n+1\right)=\hat{w}\left(n+1\right)-\hat{w}\left(n\right)$$

(2) $${\hat{w}}^{H}\left(n+1\right)x\left(n\right)=d\left(n\right)$$

where the superscript H stands for conjugate transpose.

We use the method of Lagrange multipliers. For the general case of complex data, the cost function is estimated as follows:

(3) J(n)=‖δw^(n+1)‖2+Re[λ∗(d(n)−w^H(n+1)x(n))]

where λ is the complex Lagrange multiplier and ^∗^ is the complex conjugate; Re[⋅] represents the operation of taking the natural part, and the constraint’s contribution to the cost function is real-valued; ${\left\|\delta \hat{w}\left(n+1\right)\right\|}^{2}$ represents the square operation of the Euclidean norm, and the result is also real-valued. Thus, the cost function J(n) is a real-valued quadratic function $\hat{w}\left(n+1\right)$ and can be expressed as follows:

(4) J(n)=(w^(n+1)−w^(n))H(w^(n+1)−w^(n))+Re[λ∗(d(n)−w^H(n+1)x(n))]

In order to find the most updated weight vector with the smallest cost function J(n), the following steps are taken:

Take the derivative of the cost function J(n) with respect to ${\hat{w}}^{H}\left(n+1\right)$.

(5) $$\frac{\partial J\left(n\right)}{\partial {\hat{w}}^{H}\left(n+1\right)}=2\left(\hat{w}\left(n+1\right)-\hat{w}\left(n\right)\right)-\lambda^{*}x\left(n\right)$$

Set it to zero, and the optimal solution is

(6) $$\hat{w}\left(n+1\right)=\hat{w}\left(n\right)+\frac{1}{2}\lambda^{*}x\left(n\right)$$

Bring (6) into (2) and solve for the unknown multiplier λ.

(7) $$\lambda =\frac{2e\left(n\right)}{{\left\|x\left(n\right)\right\|}^{2}}$$

(8) $$e\left(n\right)=d\left(n\right)-{\hat{w}}^{H}\left(n\right)x\left(n\right)$$

From (6) and (7),

(9) $$\delta \hat{w}\left(n+1\right)=\hat{w}\left(n+1\right)-\hat{w}\left(n\right)=\frac{1}{{\left\|x\left(n\right)\right\|}^{2}}x\left(n\right)e^{*}\left(n\right)$$

To control the incremental change of the tap weight vector from one adaptive loop to the next without changing the direction of the vector, a positive real scale factor $\tilde{\mu }$ is introduced. The increment is defined as follows:

(10) $$\delta \hat{w}\left(n+1\right)=\hat{w}\left(n+1\right)-\hat{w}\left(n\right)=\frac{\tilde{\mu }}{{\left\|x\left(n\right)\right\|}^{2}}x\left(n\right)e^{*}\left(n\right)$$

(11) $$\hat{w}\left(n+1\right)=\hat{w}\left(n\right)+\frac{\tilde{\mu }}{{\left\|x\left(n\right)\right\|}^{2}}x\left(n\right)e^{*}\left(n\right)$$

When the tap vector is small, the smaller square norm ${\left\|x\left(n\right)\right\|}^{2}$ has to be divided by $\tilde{\mu }$, which may cause numerical difficulties. To overcome this problem, (11) is revised to read as follows:

(12) $$\hat{w}\left(n+1\right)=\hat{w}\left(n\right)+\frac{\tilde{\mu }}{{\left\|x\left(n\right)\right\|}^{2}+c}x\left(n\right)e^{*}\left(n\right)$$

where *c* is a small positive integer.

The traditional NLMS algorithm uses a fixed step size, which cannot be dynamically adjusted according to the characteristics of the current signal and has poor adaptability to the signal’s dynamic characteristics. The VSS-NLMS algorithm can solve this problem very well [24]. We use the step-size update equation in the literature [31], as shown in Equation (13):

(13) $$\tilde{\mu }\left(n\right)=\beta \left(1-\exp\left(-\alpha {\left|e\left(n\right)\right|}^{2}\right)\right)$$

where $\tilde{\mu }\left(n\right)$ is the step size of the n iteration; α is a constant to control the step-size range; and β is another constant to adjust the speed of the step length. Usually, α>0 and β>0.

In the VSS-NLMS algorithm, the expected signal d(n) plays an important role, and the selection of this signal will directly affect the convergence speed, stability, and filtering effect. However, in practical applications, we usually do not know the real expected signal of a noisy signal. Currently, an input signal or its variant is often used as the expected signal of the filtering system [24].

**Figure 2:**
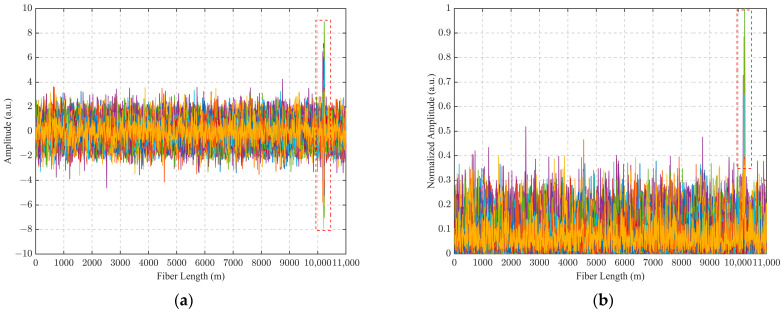
Vibration signals acquired by the Φ-OTDR system: (**a**) a 100 Hz vibration signal was applied from 10.19 to 10.24 km; (**b**) signals taken in absolute value after applying 100 Hz vibration normalization at 10.19 to 10.24 km.

**Figure 3:**
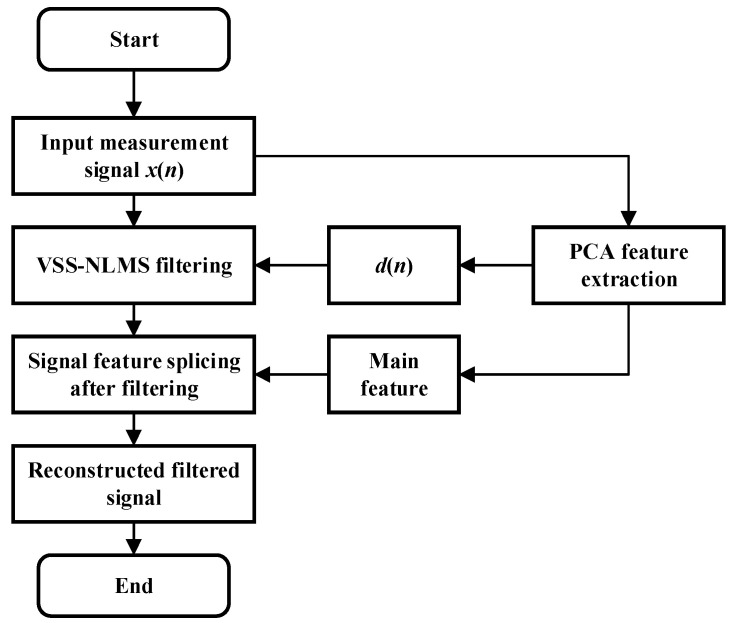
Flowchart of the PCA-VSS-NLMS algorithm.

The vibration signals of the sensing fiber obtained by the Φ-OTDR system are shown in Figure 2. Among them, the detection fiber was 11.22 km, the PZT parameters were set, the output waveform was set to a sine wave, the amplitude value was the peak-to-peak value (VPP) of the signal set to 10 V. We designed a PCA-VSS-NLMS filtering algorithm, which uses PCA to extract the position information features of the sensing fiber instead of the expected signals in VSS-NLMS and then splices the extracted main features with the data processed via PCA-VSS-NLMS filtering. The detailed algorithm flow is shown in Figure 3.

The sample matrix X is represented as follows:

(14) $$X=\left[\begin{array}{ccc} x_{11} & \cdots & x_{1l}\\ \cdots & \cdots & \cdots \\ x_{t1} & \cdots & x_{tl} \end{array}\right]$$

where t represents the sampling time, l represents the spatial position of the sampling point, and $x_{tl}$ represents the amplitude of the phase signal at the l point at time t.

The sample matrix is normalized as follows:

(15) $$X_{ij}=\frac{x_{ij}-{\bar{x}}_{j}}{s_{i}}\;\left(i=1,2,\ldots ,t;j=1,2,\ldots ,l\right)$$

(16) $$s_{j}^{2}=\frac{\sum_{i=1}^{t}\left(x_{ij}-{\bar{x}}_{j}\right)}{t-1}\;,\;{\bar{x}}_{j}=\frac{\sum_{i=1}^{t}x_{ij}}{t}$$

where $X_{ij}$ represents the standardized data matrix, $x_{ij}$ represents the standardized value of the jth index of the ith sample, $s_{i}$ represents the standard deviation, $s_{j}^{2}$ represents the variance, and ${\bar{x}}_{j}$ represents the mean value.

The covariance matrix Σ is calculated as follows:

(17) $$\Sigma =\frac{1}{t}{\left(X_{ij}\right)}^{T}\left(X_{ij}\right)$$

The eigenvalue decomposition is performed on the given covariance matrix Σ. This will yield eigenvalues $r_{1},r_{2},\ldots ,r_{n}$ and their corresponding eigenvectors $v_{1},v_{2},\ldots ,v_{n}$, where *n* is the number of features in the data. The eigenvalues are arranged in descending order. The top *k* eigenvalues are selected along with their corresponding eigenvectors. These eigenvectors will form the columns of the projection matrix W. Let us denote the selected eigenvectors as $v_{1},v_{2},\ldots ,v_{k}$. The selected eigenvectors are then arranged as columns in the projection matrix W. Thus, $W=\left[v_{1},v_{2},\ldots ,v_{k}\right]$, where $v_{1},v_{2},\ldots ,v_{k}$ are the top *k* eigenvectors obtained from the eigenvalue decomposition. The original data matrix X is multiplied by the projection matrix W, resulting in the following:

(18) $$X_{pcanew}=X\cdot W$$

where $X_{pcanew}$ denotes the newly generated data matrix that can be used as the expected signal of the principal component analysis expectation variable step normalized least mean square (PCA-d-VSS-NLMS) filter.

In the Φ-OTDR system, we pay more attention to the amplitude change of the phase signal. To further improve the SNR of the signal, we perform feature splicing of the feature data points in each row of $X_{pcanew}$ with the filtered matrix $X_{pca-d-vss-nlms}$ row by row.

(19) $$X_{pcanew}=\left[\begin{array}{cccc} x_{pn1\;1} & x_{pn1\;2} & \cdots & x_{pn1\;l}\\ x_{pn2\;1} & x_{pn2\;2} & \cdots & \cdots \\ \vdots & \vdots & & \vdots \\ x_{pnt\;1} & x_{pnt\;2} & \cdots & x_{pnt\;l} \end{array}\right]$$

(20) $$X_{pca-d-vss-nlms}=\left[\begin{array}{cccc} x_{pdvn1\;1} & x_{pdvn1\;2} & \cdots & x_{pdvn1\;l}\\ x_{pdvn2\;1} & x_{pdvn2\;2} & \cdots & \cdots \\ \vdots & \vdots & & \vdots \\ x_{pdvnt\;1} & x_{pdvnt\;2} & \cdots & x_{pdvnt\;l} \end{array}\right]$$

Assuming the data at the position of the 800th column of the 1st row in the $X_{pcanew}$ matrix are the feature data points, the data at $x_{pn1\;800}$ are replaced with the data at $x_{pdvn1\;800}$ to obtain the final filtered matrix $X_{pca-vss-nlms}$.

Filter feature data by setting a threshold to remove noisy data with small absolute values of amplitude. Only the peaks exceeding the set threshold are retained as feature data. In this paper, we utilize the statistical properties of noise to set the adaptive threshold for feature point extraction, assuming that the noise obeys a Gaussian distribution, and calculate the adaptive threshold:

(21) $$T=\mu_{n}+k_{t}\cdot \sigma_{n}$$

where T is the adaptive threshold, $\mu_{n}$ is the estimated noise mean, $\sigma_{n}$ is the estimated noise standard deviation, and $k_{t}$ is the scale factor. In this paper, $k_{t}=2$ is set.

## 3. Experimental Section

### 3.1. Simulation Experiments and Results

The SNR calculation is defined as follows:

(22) $$SNR=20\log_{10}\left(\frac{A_{signal}}{A_{nosie}}\right)$$

where $A_{signal}$ and $A_{nosie}$ are the signal and noise amplitudes, respectively, and we calculate the root mean square of the noise signal as $A_{nosie}$.

**Figure 4:**
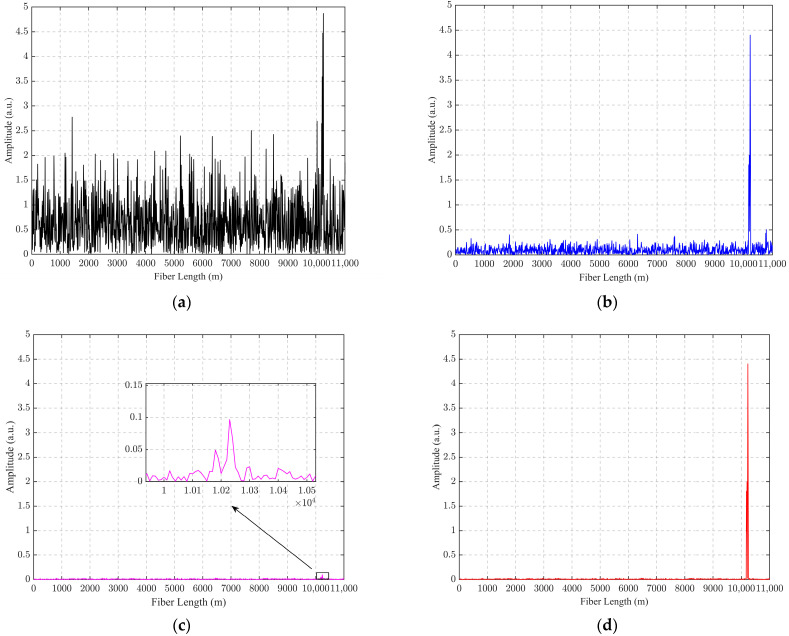
Plot of the effect of each filtering process of PCA-VSS-NLMS at 0.07 s with 100 Hz vibration applied from 10.19 km to 10.24 km: (**a**) Signal to be filtered; (**b**) PCA-filtered signals; (**c**) PCA-d-VSS-NLMS-filtered signals; (**d**) PCA-VSS-NLMS-filtered signals.

According to Figure 4 brought into Equation (19), the SNR for each stage of the proposed algorithm in this paper is calculated as:

(23) $$\begin{array}{l} SNR_{raw}=20\log_{10}\left(\frac{A_{signal\_raw}}{A_{nosie\_raw}}\right)=15.25\;\mathrm{dB}\\ SNR_{pca}=20\log_{10}\left(\frac{A_{signal\_pca}}{A_{nosie\_pca}}\right)=23.41\;\mathrm{dB}\\ SNR_{pca-d-vss-nlms}=20\log_{10}\left(\frac{A_{signal\_pdvn}}{A_{nosie\_pdvn}}\right)=1.36\;\mathrm{dB}\\ SNR_{pca-vss-nlms}=20\log_{10}\left(\frac{A_{pca-vss-nlms}}{A_{pca-vss-nlms}}\right)=30.33\;\mathrm{dB} \end{array}$$

The SNR is improved by 8.16 dB from the original signal to PCA processing, and 15.08 dB from the original signal to PCA-VSS-NLMS processing.

**Figure 5:**
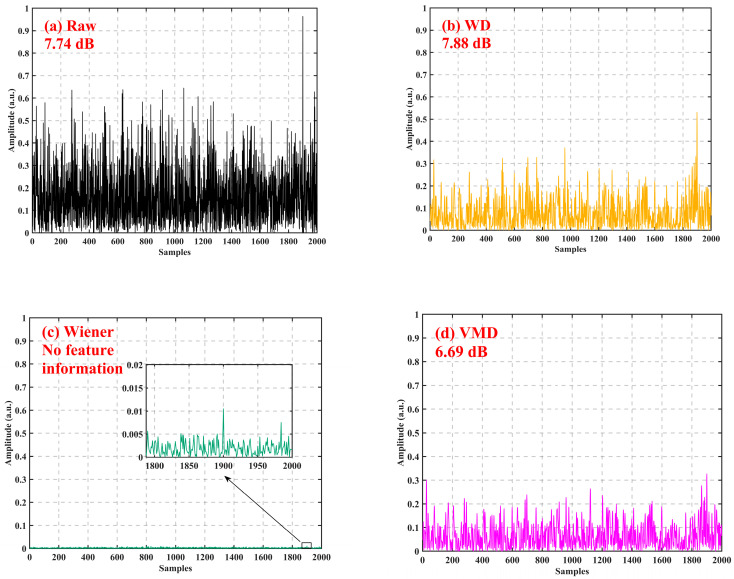

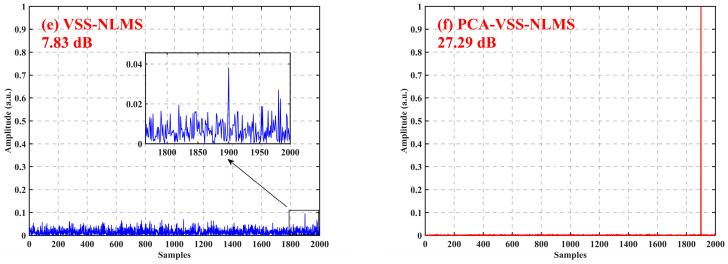
Comparison of the filtering effects of different algorithms: (**a**) signal to be filtered; (**b**) WD denoising; (**c**) Wiener denoising; (**d**) VMD denoising; (**e**) VSS-NLMS denoising; and (**f**) PCA-VSS-NLMS denoising.

We performed simulation experiments to verify the effectiveness of the algorithm and compared its performance with other algorithms. First, the unit pulse signal was used as the expected signal, with 2000 sampling points, 0.9 at the 1900th sampling point and 0 at the rest. Gaussian white noise was added to the unit pulse signal as the filter input signal, as shown in Figure 5a. The SNR was 7.74 dB. 

When comparing the performance of various filtering algorithms, the wavelet basis function of the WD algorithm was sym8, the wavelet order was 2, and the SNR after filtering was 7.78 dB, as shown in Figure 5b. The Wiener algorithm could not find feature points after filtering, as shown in Figure 5c. In the VMD algorithm, the penalty parameter was 24, the number of modal components was 4, and the SNR after filtering was 6.69 dB, as shown in Figure 5d. In the VSS-NLMS filtering algorithm, the step-size update equation α was 8, β was 0.01, the filter order was 100, and the SNR after filtering was 7.83 dB, as shown in Figure 5e. In the proposed PCA-VSS-NLMS filtering algorithm, the step-size update equation α was 8; β was 0.01; the filter order was 100; the number of retained principal components was 6; k=1 feature vectors were selected as the principal components, and the selected feature vectors were arranged into a projection matrix by column; and the SNR after filtering was 27.29 dB, as shown in Figure 5f. To further verify the effectiveness of the algorithm, Gaussian white noise with different SNRs was added to the unit pulse signal to compare the SNR enhancement effects of the WD, Wiener, VMD, VSS-NLMS, and PCA-VSS-NLMS algorithms, as shown in Table 1.

The results presented in Table 1 demonstrate the impact of various filtering algorithms on noise SNRs. The data indicate that the PCA-VSS-NLMS algorithm outperforms other algorithms in terms of SNR enhancement across different noise levels. In particular, at an SNR of −1.23 dB, the PCA-VSS-NLMS algorithm successfully preserves a signal with an SNR of 30.68 dB, while other algorithms fail to do so. These findings suggest that the filtering algorithm introduced in this study effectively enhances the SNR and mitigates noise interference.

### 3.2. Experimental Setup and Results

**Figure 6:**
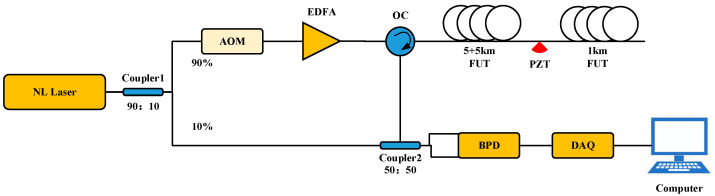
Schematic diagram of the Φ-OTDR system.

Figure 6 shows the construction of the Φ-OTDR system. A laser with a narrow linewidth of 1550.12 nm central wavelength and a 3 kHz linewidth is used to split the light into two channels of 90% and 10% using coupler-1. Through an acousto-optic modulator (AOM), 90% of the light is converted into a 200 MHz frequency shifted optical pulse, which is then amplified by an erbium-doped fiber amplifier (EDFA) before entering an optical circulator (OC). At coupler two, the RBS light from the sensing fiber interferes with the local light to produce beat light. The output signal from the beat light is detected by a balanced photodetector (BPD), which transfers it to the computer via a data acquisition card (DAQ) for phase signal extraction.

We use the I/Q quadrature demodulation method proposed in the literature [20] to demodulate Rayleigh’s backscattered light amplitude and phase signals.

The beat frequency signals acquired by the Φ-OTDR system are as follows:

(24) $$P\left(t\right)=E_{R}\left(t\right)E_{LO}\left(t\right)\cos\left[2\pi \Delta ft+\Phi \left(t\right)+\Phi_{0}\right]$$

where $E_{R}$ is the backward Rayleigh scattered light, $E_{LO}$ is the reference light, Δf is the AOM frequency shift, Φ(t) is the phase change due to the vibration signal, and $\Phi_{0}$ is the phase change due to the noise signal.

(25) $$y_{1}=\cos\left(2\pi \Delta ft+\Phi_{r}\right)$$

(26) $$y_{2}=\sin\left(2\pi \Delta ft+\Phi_{r}\right)$$

where $\Phi_{r}$ is the phase noise, multiplying Equation (24) by Equations (25) and (26), respectively, yields:

(27) $$\begin{array}{c} I\left(t\right)=E_{R}\left(t\right)E_{LO}\left(t\right)\cos\left[2\pi \Delta ft+\Phi \left(t\right)+\Phi_{0}\right]\cos\left(2\pi \Delta ft+\Phi_{r}\right)\\ \;\;\;\;\;\;\;\;\;\;\;\;\;\;\;\;\;\;\;\;\;\;\;\;\;\;\;\;\;\;\;\;\;\;=\frac{1}{2}E_{R}\left(t\right)E_{LO}\left(t\right)\left[\cos\left[4\pi \Delta ft+\Phi \left(t\right)+\Phi_{0}+\Phi_{r}\right]+\cos\left[\Phi \left(t\right)+\Phi_{0}-\Phi_{r}\right]\right] \end{array}$$

(28) $$\begin{array}{c} Q\left(t\right)=E_{R}\left(t\right)E_{LO}\left(t\right)\cos\left[2\pi \Delta ft+\Phi \left(t\right)+\Phi_{0}\right]\sin\left(2\pi \Delta ft+\Phi_{r}\right)\\ \;\;\;\;\;\;\;\;\;\;\;\;\;\;\;\;\;\;\;\;\;\;\;\;\;\;\;\;\;\;\;\;\;\;=\frac{1}{2}E_{R}\left(t\right)E_{LO}\left(t\right)\left[\cos\left[4\pi \Delta ft+\Phi \left(t\right)+\Phi_{0}+\Phi_{r}\right]-\sin\left[\Phi \left(t\right)+\Phi_{0}-\Phi_{r}\right]\right] \end{array}$$

The orthogonal signals I(t) and Q(t) are obtained after low-pass filtering.

(29) $$I\left(t\right)=\frac{1}{2}E_{R}\left(t\right)E_{LO}\left(t\right)\cos\left[\Phi \left(t\right)+\Phi_{0}-\Phi_{r}\right]$$

(30) $$Q\left(t\right)=-\frac{1}{2}E_{R}\left(t\right)E_{LO}\left(t\right)\sin\left[\Phi \left(t\right)+\Phi_{0}-\Phi_{r}\right]$$

(31) $$A_{s}\propto \sqrt{I{\left(t\right)}^{2}+Q{\left(t\right)}^{2}}$$

(32) $$\Phi \left(t\right)=-\arctan\frac{Q\left(t\right)}{I\left(t\right)}$$

where $\Phi_{0}-\Phi_{r}$ is the phase noise, $A_{s}$ is the signal amplitude, and Φ(t) is the phase change due to the vibration signal.

**Figure 7:**
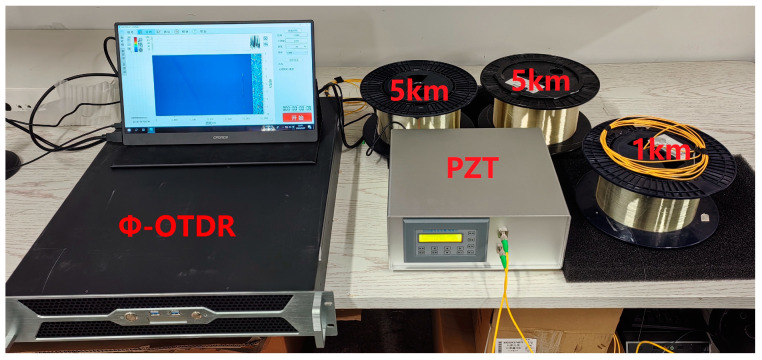
The experimental environment of the Φ-OTDR system.

**Figure 8:**
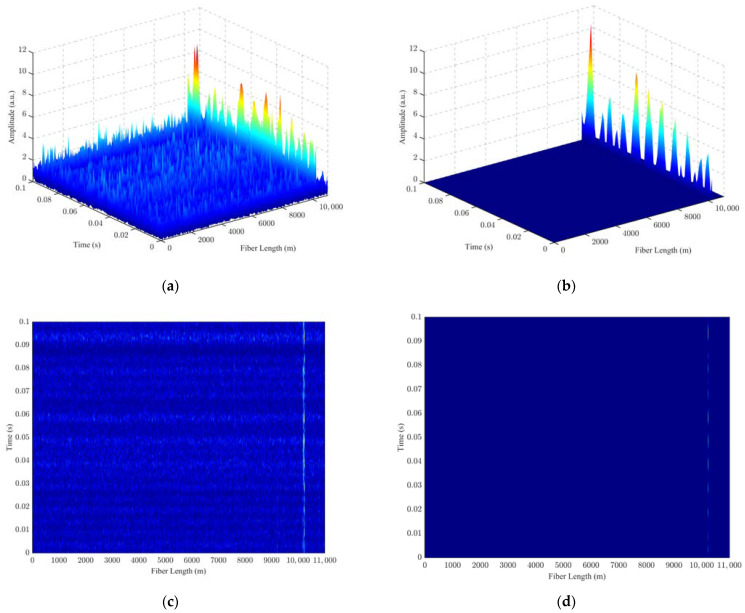
3D spatio-temporal maps of measured vibration signals: (**a**) 3D spatio-temporal map of unfiltered vibration signals at 11.22 km; (**b**) 3D spatio-temporal map of filtered vibration signals at 11.22 km PCA-VSS-NLMS; (**c**) 3D spatio-temporal map top view of unfiltered vibration signals at 11.22 km; (**d**) 3D spatio-temporal map top view of filtered vibration signals at 11.22 km PCA-VSS-NLMS.

We connected the Φ-OTDR system to an 11 km G 652D single-mode fiber. The experimental setup is shown in Figure 7. The optical outlet of the Φ-OTDR system was connected to the first 5 km disc fiber, whose end was fused to the second 5 km disc fiber; the second 5 km disc fiber was connected to the PZT device INPUT, and then the PZT device OUTPUT was connected to a 1 km disc fiber. The actual length of the fiber measured by the OTDR was 11.22 km, the PZT inner winding used a 50 m fiber, and the vibration position was 10.19–10.24 km. The Φ-OTDR system parameters were set as follows: the detection range was set to 11 km, and the spatial resolution was set to 10 m. The PZT parameters are set as follows: the output waveform is set as a sinusoidal waveform, and the amplitude value is the peak-to-peak value of the signal VPP, which is set to 10 V. The frequencies of 100 Hz, 200 Hz, 300 Hz, 400 Hz, 500 Hz, 600 Hz, 700 Hz, 800 Hz, and 900 Hz were set to compare the effects of the filtering algorithms. The experimental results of the PCA-VSS-NLMS algorithm proposed in this paper are shown in Figure 8. System components, PZT, are procured from Nanjing Fiber Photonics Technology Co., Ltd, Nanjing, Jiangsu Province, China. It was accessed on 12 June 2024 at http://www.fib-tech.com/.

The experiment was repeated 10 times for each fixed-frequency signal under the same conditions, and the average SNR at 0.07 s was recorded. The comparison data of filtering effects are shown in Table 2. As can be seen from Table 2, which is much higher than that of the other filtering algorithms.

A three-dimensional (3D) spatio-temporal diagram was drawn for the PZT vibration signal frequency at 100 Hz, as shown in Figure 8. In this figure, Figure 8a is the 3D spatio-temporal diagram of the unfiltered measured data; Figure 8b shows the 3D spatio-temporal diagram of the filtered data after applying the PCA-VSS-NLMS algorithm; Figure 8c shows the top view of the 3D spatio-temporal diagram of the unfiltered measured data; and Figure 8d shows the top view of the 3D spatio-temporal diagram of the filtered data after applying the PCA-VSS-NLMS algorithm. It can be seen from Figure 8 that the vibration position is consistent with the actual position at 10.19–10.24 km. The proposed PCA-VSS-NLMS algorithm can effectively filter out the background noise and highlight the characteristics of vibration signals.

**Figure 9:**
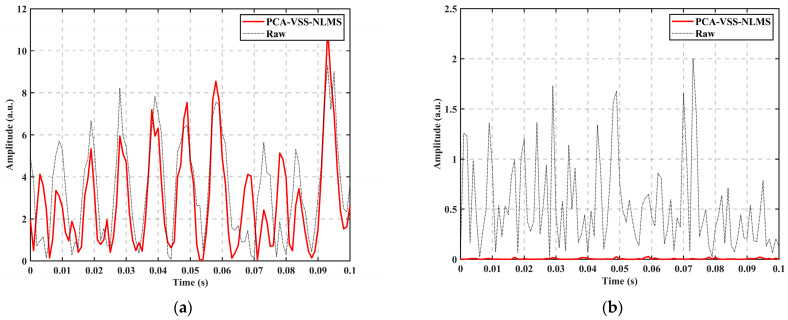
Fixed-position time-domain signal plots: (**a**) before and after filtering at 10.23 km; (**b**) before and after filtering at 10.00 km.

The time-domain signal at the fixed position in Figure 8 is selected, as shown in Figure 9. The red solid line is the curve after PCA-VSS-NLMS processing, and the black dashed line is the curve of the measured data, where Figure 9a is the comparison of the vibration signal before and after the filtering at the vibration position of PZT at 10.23 km. From Figure 9a, it can be seen that the features of the vibration signal are well preserved, and Figure 9b is the comparison before and after filtering of the background noise at 10.00 km. From Figure 9b, it can be seen that the background noise is well suppressed.

**Figure 10:**
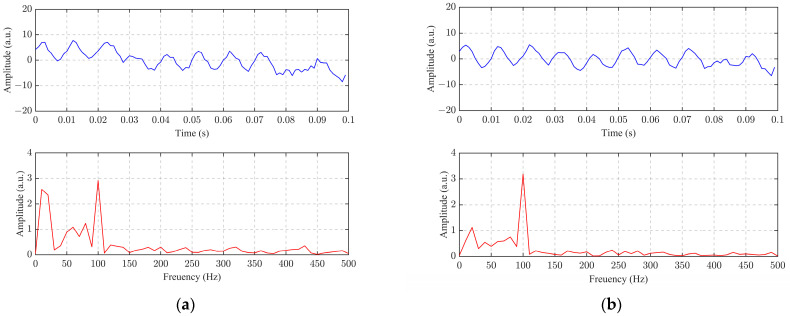
Time-frequency domain plot of PZT vibration at 100 Hz, 10.23 km from the optical fiber: (**a**) Time-frequency domain plot before filtering; (**b**) Time-frequency domain plot after filtering.

The PZT vibration frequency is 100 Hz, and the time-frequency domain diagram at 10.23 km of the fiber is shown in Figure 10, where Figure 10a is the time-frequency domain diagram before filtering; Figure 10b is the time-frequency domain diagram after filtering. A comparison of the spectra of Figure 10a and Figure 10b shows that the low-frequency component is effectively suppressed after filtering.

**Figure 11:**
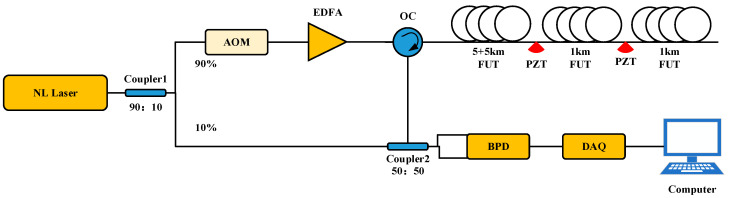
Schematic diagram of the two-point vibration experiment of the Φ-OTDR system.

**Figure 12:**
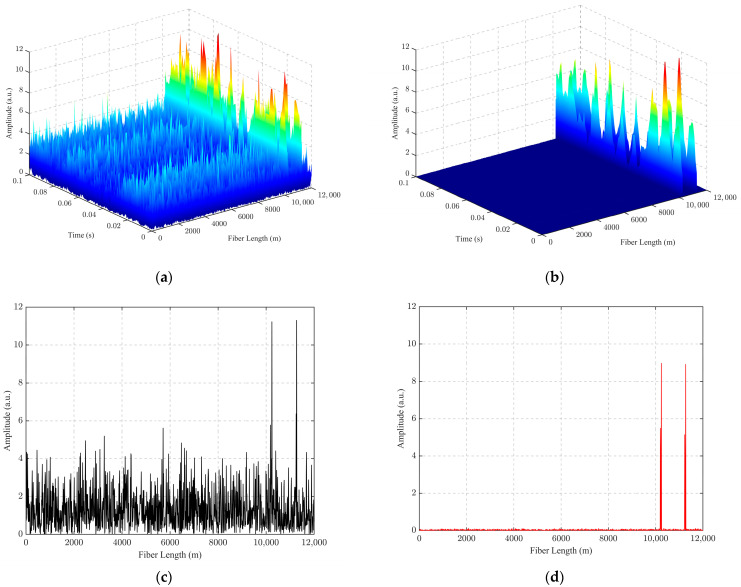
3D spatio-temporal maps of two-point vibration experiments: (**a**) 3D spatio-temporal maps of unfiltered measured data; (**b**) 3D spatiotemporal maps of PCA-VSS-NLMS-filtered data; (**c**) unfiltered signals at 0.07 s; (**d**) PCA-VSS-NLMS-filtered signals at 0.07 s.

For the dual-point vibration experiment, the Φ-OTDR system was connected to a 12 km G 652D single-mode fiber. The schematic diagram of the dual-point position vibration Φ-OTDR system is shown in Figure 11. The actual length of the fiber measured by OTDR is 12.25 km, and the Φ-OTDR system and PZT parameter settings are the same as in Figure 6. A 100 Hz frequency vibration is applied at 10.19–10.24 km and a 100 Hz frequency vibration is applied at 12.20–11.25 km, respectively. The experimental results of the PCA-VSS-NLMS algorithm proposed in this paper are shown in Figure 12, where Figure 12a is the 3D spatio-temporal map of the unfiltered measurement data, Figure 12b is the 3D spatial-temporal plot of the PCA-VSS-NLMS filtered data, Figure 12c is the unfiltered vibration signal at 0.07 s, and Figure 12d is the PCA-VSS-NLMS filtered vibration signal at 0.07 s. The 100 Hz vibration point SNR was raised from 9.05 dB to 22.26 dB, a mention of 13.21 dB. The SNR of the second 100 Hz vibration point increased from 8.57 dB to 22.23 dB, an improvement of 13.66 dB.

**Figure 13:**
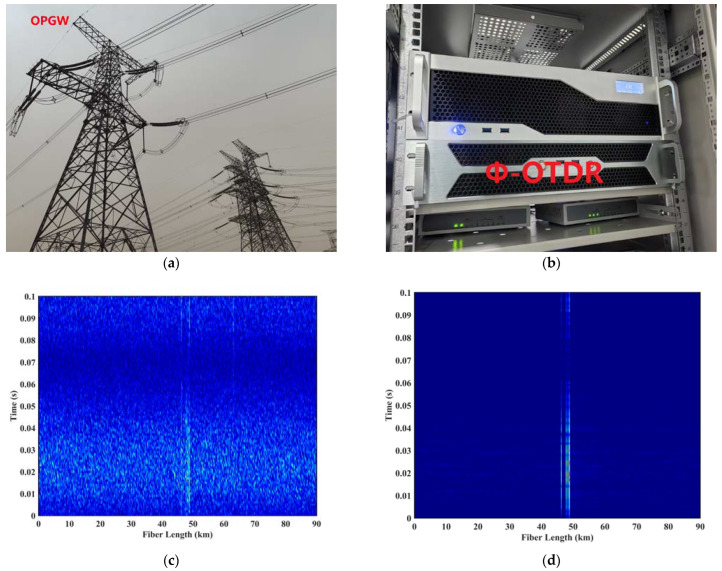
Installation and analysis results: (**a**) schematic diagram of the OPGW cable’s location; (**b**) installation diagram of the Φ-OTDR system in the station; (**c**) top view of the 3D spatio-temporal diagrams of unfiltered measured vibration data from the 90 km OPGW fiber-optic cable; and (**d**) top view of the 3D spatio-temporal diagrams of vibration data filtered by PCA-VSS-NLMS from the 90 km OPGW fiber-optic cable.

To further verify the practical application effect of the algorithm proposed in this study, on 14 April 2024, we embedded the PCA-VSS-NLMS algorithm into the Φ-OTDR system and installed the system in the communication room of the 500 kv Station A of Tongliao City, Inner Mongolia Province of China, as part of the state grid. We measured a 90 km optical fiber composite overhead ground wire (OPGW) as the optical cable line. The Φ-OTDR system parameters were set as follows: the detection range was set to 90 km, and the spatial resolution was set to 100 m. The installation and analysis results are shown in Figure 13.

Where Figure 13a is a schematic diagram of the OPGW cable’s location; Figure 13b is the installation diagram of the Φ-OTDR system in the station; Figure 13c shows the top view of the 3D spatial spectrum of unfiltered measured vibration data from the 90 km OPGW fiber-optic cable; and Figure 13d shows the top view of the 3D spatial spectrum of vibration data filtered by PCA-VSS-NLMS from the 90 km OPGW cable. As shown in Figure 13c,d, the proposed filtering algorithm can effectively filter out the background noise and highlight the vibration signal position. As can be seen in Figure 13d, there is a strong vibration signal at a distance of 46–49 km, which can be viewed as a multi-point vibration. It was verified that the 46–49 km line was in a level 2 dance zone and that the meteorological data for that day were a southerly wind at level 5.

## 4. Conclusions

In this study, we enhanced the position information SNR of the Φ-OTDR system by introducing a filtering algorithm based on PCA-VSS-NLMS. The mathematical foundation of the PCA-VSS-NLMS algorithm was elucidated, and its effectiveness was established through simulation experiments. The results from the simulation experiments show that the PCA-VSS-NLMS algorithm achieves a significant improvement in SNR, reaching up to 30.68 dB when the initial SNR is only −1.23 dB. It outperforms existing algorithms such as WD, Wiener, VMD, and VSS-NLMS, highlighting the potential of the proposed algorithm in enhancing the performance of the Φ-OTDR system. The PCA-VSS-NLMS algorithm was embedded into the built Φ-OTDR system, an 11.22 km fiber was measured, and PZT was added at 10.19–10.24 km to impose multiple sets of fixed-frequency disturbances. The experimental results show that the SNR of the vibration signal is 8.77 dB at 100 Hz and 0.07 s, and the SNR is improved to 26.17 dB after PCA-VSS-NLMS filtering; thus, the SNR is improved by 17.40 dB. In addition, we carried out practical application measurements to monitor the vibration of a 90 km OPGW as an optical cable line of the 500 kv Station A in Tongliao City, Inner Mongolia Province of China, as part of the state grid, effectively reducing the background noise. The measurement results are consistent with the actual situation. The proposed algorithm can improve the SNR of the Φ-OTDR system’s position information without changing the existing hardware conditions and provides a new scheme for the detection and recognition of long-distance vibration signals.

## Figures and Tables

**Table 1:** Comparison of the filtering effects of SNR algorithms with different noise signals.

	Noisy Signal SNR							
Filtering Algorithm	−1.23 dB	4.88 dB	7.74 dB	10.24 dB	12.51 dB	14.96 dB	17.19 dB	19.82 dB
WD	No signal	No signal	7.88	12.72	15.36	17.85	19.87	22.73
Wiener	No signal	No signal	No signal	8.32	10.41	13.23	16.79	21.64
VMD	No signal	No signal	6.69	6.46	9.67	12.41	14.18	17.18
VSS-NLMS	No signal	5.04	7.86	10.39	12.85	15.09	17.24	19.78
PCA-VSS-NLMS	30.68	26.65	27.29	27.89	28.49	29.60	30.62	31.55

**Table 2:** Comparison of filtering effects of different filtering algorithms at different frequencies.

Filtering Algorithm	100 Hz SNR/dB	200 Hz SNR/dB	300 Hz SNR/dB	400 Hz SNR/dB	500 Hz SNR/dB	600 Hz SNR/dB	700 Hz SNR/dB	800 Hz SNR/dB	900 Hz SNR/dB
Unfiltered	8.77	9.36	9.93	10.03	11.16	9.27	7.70	10.44	7.91
WD	11.47	10.40	12.18	11.79	11.82	12.67	10.88	13.59	10.36
Wiener	6.02	7.69	7.97	8.17	9.31	7.52	5.07	9.44	5.28
VMD	6.61	6.34	7.34	8.76	8.37	9.46	6.08	9.24	8.57
VSS-NLMS	8.92	9.03	9.95	9.95	10.92	9.19	7.58	10.20	7.27
PCA-VSS-NLMS	26.17	26.40	25.52	24.52	26.92	25.39	28.35	26.01	26.92

## Data Availability

The data presented in this study are available from the corresponding author upon request. The data are not publicly available due to potential commercial values.
